# Preventive antibiotic treatment of calves: emergence of dysbiosis causing propagation of obese state‐associated and mobile multidrug resistance‐carrying bacteria

**DOI:** 10.1111/1751-7915.13496

**Published:** 2019-10-30

**Authors:** Dorota A. Dobrzanska, Matthew T. F. Lamaudière, Jessica Rollason, Lauren Acton, Michael Duncan, Sharon Compton, John Simms, Gareth D. Weedall, Igor Y. Morozov

**Affiliations:** ^1^ Centre for Sport, Exercise and Life Sciences Coventry University Coventry UK; ^2^ School of Life Sciences Coventry University Coventry UK; ^3^ Moreton Morrell College Farm The Warwickshire College Warwickshire CV35 9BL UK; ^4^ School of Natural Sciences and Psychology Liverpool John Moores University Liverpool UK

## Abstract

In agriculture, antibiotics are used for the treatment and prevention of livestock disease. Antibiotics perturb the bacterial gut composition but the extent of these changes and potential consequences for animal and human health is still debated. Six calves were housed in a controlled environment. Three animals received an injection of the antibiotic florfenicol (Nuflor), and three received no treatment. Faecal samples were collected at 0, 3 and 7 days, and bacterial communities were profiled to assess the impact of a therapy on the gut microbiota. Phylogenetic analysis (16S‐rDNA) established that at day 7, antibiotic‐treated microbiota showed a 10‐fold increase in facultative anaerobic *Escherichia spp*, a signature of imbalanced microbiota, dysbiosis. The antibiotic resistome showed a high background of antibiotic resistance genes, which did not significantly change in response to florfenicol. However, the maintenance of *Escherichia coli* plasmid‐encoded quinolone, *oqxB* and propagation of *mcr*‐2, and colistin resistance genes were observed and confirmed by Sanger sequencing. The microbiota of treated animals was enriched with energy harvesting bacteria, common to obese microbial communities. We propose that antibiotic treatment of healthy animals leads to unbalanced, disease‐ and obese‐related microbiota that promotes growth of *E. coli* carrying resistance genes on mobile elements, potentially increasing the risk of transmission of antibiotic resistant bacteria to humans.

## Graphical abstract

Antibiotic treatment leads to expansion of facultative anaerobic *Escherichia* spp. (proteobacteria) in calves, a signature of imbalanced microbiota, dysbiosis. This in turn enhances a risk of the growth of bacteria that carry highly mobile clinically relevant resistances that can be transmitted to humans.

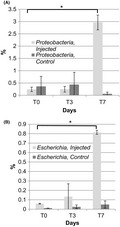

## Introduction

The intestinal microbiota is critical for homeostasis in humans and animals and represents a natural reservoir of antibiotic resistance genes (Salyers *et al.*, 2004; Dethlefsen *et al.*, 2007; Sommer *et al.*, 2009; Allen *et al.*, 2010). Antibiotics (AB) are commonly used in livestock farming to prevent and treat infections as well as in developing countries to support growth, despite promoting the rapid development of multidrug‐resistant pathogens including those resistant to ‘antibiotics of last resort’, such as colistin (Cabello, 2006; McEwen, 2006; Martínez, 2008; Hasman *et al.*, 2015; Liu *et al.*, 2016; Paterson and Harris, 2016). Also, it has long been thought that such treatments directly select for bacteria resistant to the prescribed antibiotic, leading to emergence of resistance determinates specific to the administrated antibiotic (e.g. Hayden *et al.*, 2005; Sanchez Garcia *et al.*, 2010; Endimiani *et al.*, 2011). However, AB treatment appears to cause the more general imbalance of the microbiota dysbiosis that can have additional effects, including a disproportional increase in abundance of specific bacteria which may carry mobile elements that contain multidrug resistance genes (Barlow, 2009; Shin *et al.*, 2015) and/or being associated with obesity.

An overlooked aspect of the widespread use of antibiotics is how they affect the composition of the gut microbiota with relation to the host wellbeing. There is an ever‐growing body of evidence to support the role of the gut flora in human and animal health and disease (Holmes *et al.*, 2011). A balanced intestinal microbiota, generally dominated by obligate anaerobes due to the hypoxic nature of the gut, is vital in maintaining homeostasis, energy metabolism and supplying essential biomolecules that are not synthesized by the host (Zoetendal *et al.*, 2004; Krajmalnik‐Brown *et al.*, 2012). AB treatment is known to perturb the intestinal microbiota, in part due to elevating the oxygen level in the colon (Meynell, 1963). AB treatment in mice (Saito, 1961), humans (Vollaard *et al.*, 1992) and pigs (Looft *et al.*, 2012) has been associated with an expansion of facultative anaerobes, disrupting anaerobiosis and enhancing aerobic respiration. A disproportional increase in abundance of certain *Proteobacteria*, such as facultative anaerobes like *Escherichia coli* in the gut is linked to inflammation and obesity, can promote aerobic pathogen colonization and enhance permeability of the colon, compromising the ability of microbiota to maintain a balanced, protective bacterial community (Zoetendal *et al.*, 2004; Arumugam *et al.*, 2011; Holmes *et al.*, 2011; Shin *et al.*, 2015). Also, commensal facultative anaerobes may carry a wide range of genes responsible for genetic transfer, including resistance determinants to antibiotics that have not been administrated (Looft *et al.*, 2012), therefore increasing the prevalence of ‘off‐target’ antibiotic resistance. Changes in the composition of the gut microbiota have also been linked to weight gain in animals and humans (Cromwell, 2002; Angelakis *et al.*, 2012; Million *et al.*, 2013), and the effect on health however has continued to be overlooked. The bacterial gut microbiota composition of obese mammals differs from their lean counterparts, and it has been shown to be critical to both fat storage and host energy balance (Ley *et al.*, 2006; Turnbaugh *et al.*, 2006). Interestingly, an increase in body mass may occur without changes in food consumption, strongly suggesting that the microbiota in the gut is crucial to obesity and its composition influences the amount of energy derived from the diet (Bäckhed *et al.*, 2004). Hence, the obesity‐associated gut microbiota appears to be more efficient in energy extracting than a balanced bacterial counterpart. Such evidence demonstrates that effects of the liberal use of antibiotics have been long underestimated.

One of the emerging challenges with respect to AB‐resistant commensal bacteria is that they may carry mobile resistance elements that had previously been thought to be associated only with chromosomal mutations, for example resistance to colistin which has now been found to occur on mobile genetic elements (Liu *et al.*, 2016). While originally found in animals, colistin resistance has already been reported in humans, making zoonotic transmission a major public health concern. A similar pattern of resistance has emerged for fluoroquinolones. The previous use of colistin and/or fluoroquinolones in livestock to promote growth has led to the rise of the corresponding plasmid‐mediated resistance determinants in *E. coli* (Witte, 1998; Hansen *et al.*, 2004; Zhao *et al.*, 2010; Liu *et al.*, 2016; Shen *et al.*, 2016; Partridge, 2017). However, AB‐driven dysbiosis may further contribute to the expansion of commensal and pathogenic bacteria carrying a wide range of plasmid‐mediated multidrug resistances which are highly mobile and capable of self‐transmission between agricultural and clinical settings. Therefore, growth of bacteria that may be carrying resistance genes on mobile elements due to dysbiosis in the human food chain following antibiotic treatments poses significant risk to animals and humans (Shin *et al.*, 2015; Litvak *et al.*, 2017) has long been overlooked.

Here, calves housed and reared under controlled conditions were exposed to preventive antibiotic therapy florfenicol; trade name Nuflor to assess the effects on resistome and the gut microbiota. This is the first report of a molecular analysis of calf gut microbiota in response to a preventive antibiotic therapy that predisposes animals to dysbiosis and disease, promoting the growth of bacteria that carry highly mobile clinically relevant resistances that can be transmitted to humans.

## Results

### Antibiotic treatment leads to an imbalanced microbial community in the animal gut

**Figure 1:**
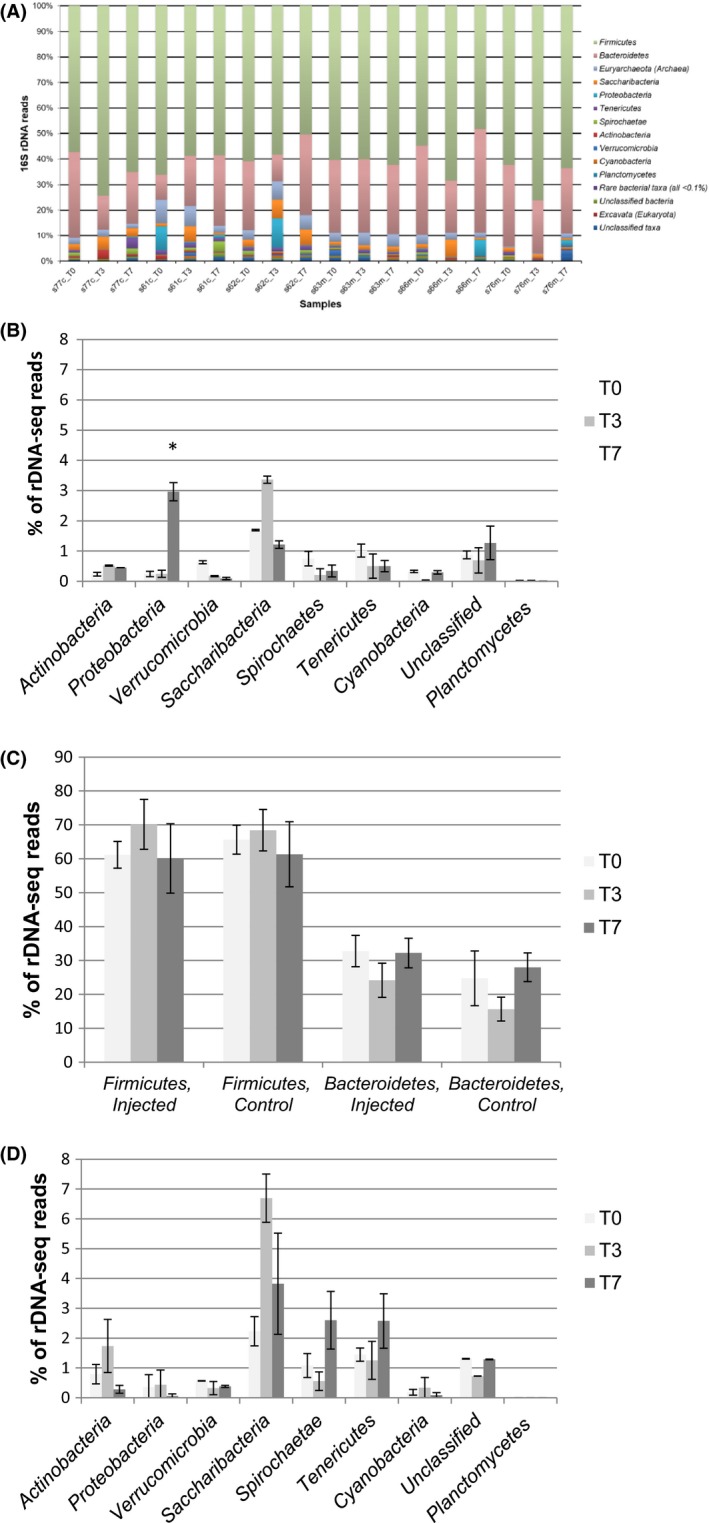
Phylogenetic analysis of the composition of faecal animal gut microbiota. (A) Phylum‐level composition of non‐medicated, control (s77c, s61c and s62c) and medicated, injected with florfenicol (s63m, s66m and s76m) faecal microbiota samples before treatment (T_0_) and after the antibiotic treatment, day 3 (T_3_) and day 7 (T_7_). The saccharolytic bacteria, *Firmicutes* and *Bacteroidetes* are the most abundant phyla, followed by *Saccharibacteria*, *Tenericutes*, *Spirochaetes* with minor contributors *Actinobacteria*, *Proteobacteria*, *Verrucomicrobia* and *Cyanobacteria*. (B) *Firmicutes* and *Bacteroidetes* phyla composition in injected and control samples. (C) Bacterial composition of less abundant phylum in injected and (D) control samples over time‐course, T_o_, T_3_ and T_7_. The y‐axis is the percentage of rDNA‐seq reads over the total number of reads. rDNA‐seq reads from three medicated and non‐medicated faecal samples were quantified and presented with the standard error (SE) of the mean (*n* = 3) at 95% confidence intervals. The asterisk (*) indicates a statistically significant difference for injected samples over the time‐course comparing with the control (non‐medicated; the intervention interaction effect between the two groups, *P* = 0.006)

**Figure 2:**
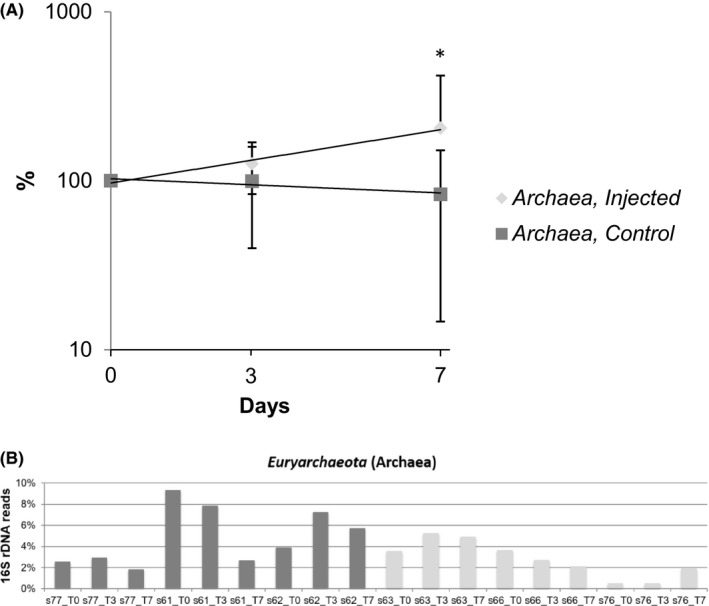
Abundance of methanogenic *Archaea* (*Methanobacteriales *spp) is significantly increased by T_7_ in response to the florfenicol treatment (*, T_o_/T_7_ *P* = 0.003 and was significantly higher comparing with non‐medicated at day 7 (*,T_7 injected_ v T_7 non‐medicated_, *P* = 0.025). (A) Time‐course over seven days for injected (medicated) and control (non‐medicated) samples was plotted. (B) *Euryarchaeota* (Archaea) composition, percentage of sequences normalized to the total number of reads shown. Error bars present SE of the mean (*n* = 3) at 95% confidence intervals

Deep sequencing of the 18 samples produced 5 578 146 sequence reads of the V3–V4 hypervariable regions of the bacterial 16S rDNA; 2 888 547 from medicated and 2 689 599 from non‐medicated calves. In each sample, up to 10 different bacterial phyla (Fig. 1A) were identified, along with methanogenic *Archaea* (Fig. 2). The predominant phyla were *Firmicutes* (64 ± 1.7%) and *Bacteroidetes* (26 ± 2.6%; Fig. 1A and 1), which have been shown to be the most abundant in human and animal guts (Eckburg *et al.*, 2005; Sun and Chang, 2014). At lower prevalence were *Saccharibacteria*, *Tenericutes*, *Spirochaetes*, *Actinobacteria*, *Proteobacteria*, *Verrucomicrobia* and *Cyanobacteria*. Except *Proteobacteria,* the overall prevalence of phyla did not significantly change in response to antibiotic treatment (Fig. 1B and 1).

**Figure 3:**
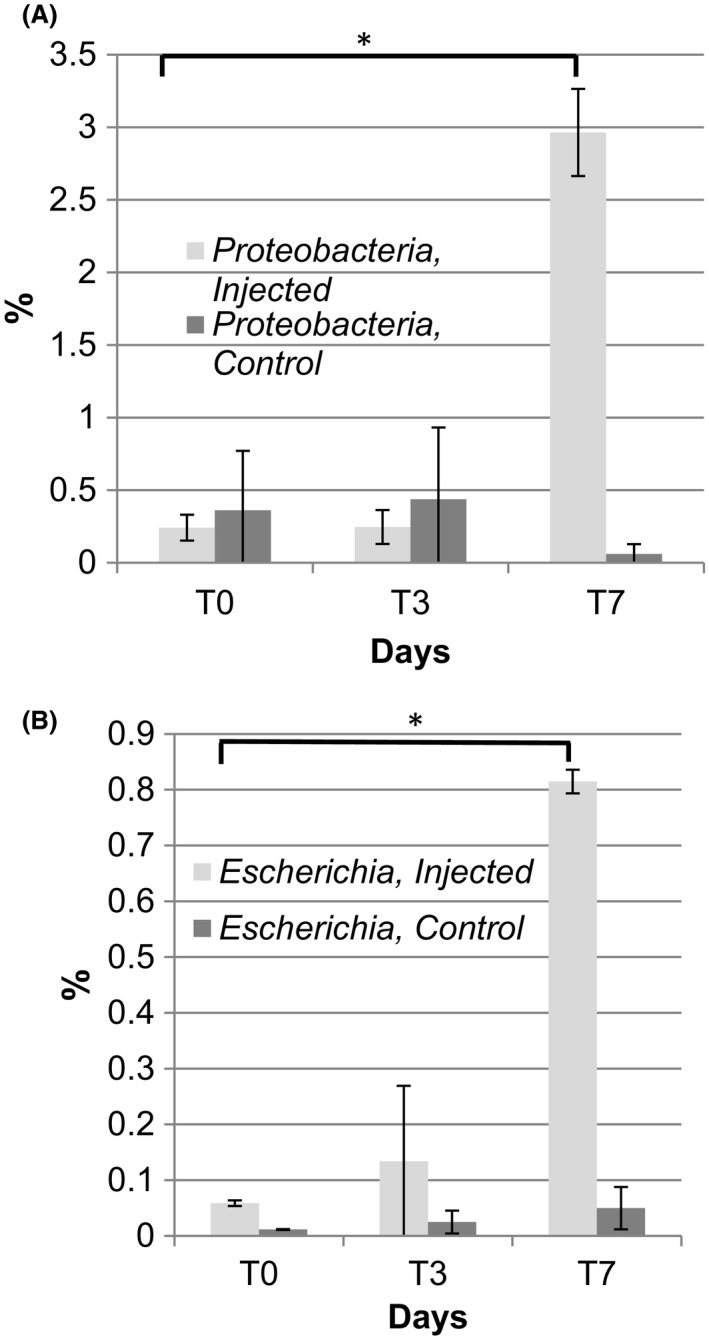
Florfenicol treatment leads to dysbiosis, an expansion of *Proteobacteria*. (A) Phylum‐level composition in the gut (*, T_o_ v T_7_ *P* = 0.01) was changed significantly in response to AB treatment (*P* = 0.017) in contrast to the control group (*P* = 0.7). A 10‐fold increase upon the AB treatment at T_7_ was observed comparing with the non‐medicated T_7_ sample (T_7 injected_ v T_7 non‐medicated_, *P* = 0.022) (B) Genus‐level composition of *Escherichia* in response to the AB treatment (*, T_o_ v T_7_, *P* ˂ 0.05 (0.011)) was significantly higher at T7 comparing with non‐medicated samples (T_7 injected_ v T_7 non‐medicated_, *P* = 0.032). No significant differences for control samples (*P* = 0.913) and between T_0_ v T_3_ for injected samples (*P* = 0.591) were observed. Details are as in Fig. 1

AB treatment changed the composition of gut microbiota through a 10‐fold rise in *Proteobacteria* by day 7 (*P* = 0.017), and this was a significant shift compared with non‐medicated samples at day 7 (*P* = 0.022; Fig. 3A). Results from repeated measures ANOVA indicated a significant intervention interaction (*P* = 0.006). Post hoc pairwise comparisons indicated that there were no significant differences in the abundance of *Proteobacteria* between T_0_, T_3_ and T_7_ for the control group. The injected group did not show an increase of the phylum from T_0_ to T_3_. There was no significant difference in the *Proteobacteria* abundance between the two groups at T_0_ and T_3_; however, there was a significant difference between the two groups at T_7_ (*P* = 0.022). Such a shift was mainly due to an expansion of the genus *Escherichia* T_0_/ v T_7_ (*P* = 0.011) in response to AB (Fig. 3B). For the injected group, there was a non‐significant trend in increased *Escherichia* from T_0_ to T_3_ (*P* = 0.591). There was no significant difference in the *Escherichia* abundance between the two groups at T_0_ and T_3_; however, there was a significant difference between the two groups at T_7_ (*P* = 0.032). The increase in *E. coli* was confirmed by semi‐quantitative PCR of the corresponding metagenomic DNAs targeting *uidA*, the gene encoding for β‐glucuronidase, a marker for *E. coli* Supplementary information (Fig. S1). A significant increase in the prevalence of *E. coli* in the gut is a distinct feature of an imbalanced microbial community (Shin *et al.*, 2015).

**Figure 4:**
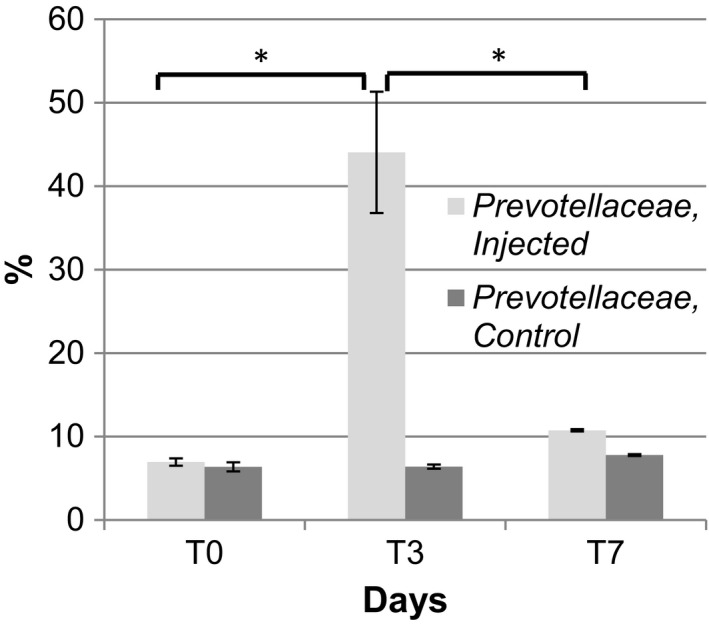
Florfenicol treatment leads to selection of obese‐related microbiota. Florfenicol treatment leads to a significant increase in relative abundance of the family *Prevotellaceae* (phylum *Bacteroidetes*; *P* = 0.013, the intervention interaction effect). Details are as in Fig. 1. Injected: *, T_o_ v T_3_ *P* = 0.029, T_3_ v T_7_ *P* = 0.022; T_3 injected_ v T_3 non‐medicated_ *P* = 0.03. No significant differences (*P* > 0.033, 0.045) for control samples were observed

The treatment led to selection of both methanogenic *Archaea* (T_0_ v T_7_ *P* = 0.003; Fig. 2) and *Prevotellaceae *spp (T_0_ vT_3_ *P* = 0.029, T_0_ v T_7_ *P* = 0.045, T_3_ v T_7_ *P* = 0.022; Fig. 4). The control group did not show significant differences in the abundance of both *Archaea* and *Prevotellaceae spp* over the time‐course. However, there were significant differences between the two groups at T_7_ (*P* = 0.025) and T_3_ (*P* = 0.03) for *Archaea* and *Prevotellaceae *spp correspondently. Repeated measures ANOVA indicated a significant intervention interaction (*P* = 0.013) for *Prevotellaceae *spp. The post hoc pairwise comparisons indicated at T_3_ point the abundance of *Prevotellaceae *spp in the injected group was significantly higher comparing with the non‐medicated one (*P* = 0.013), while there were no differences between groups at T_0_ and T_7_. For *Archaea,* there was a significant increase from T_0_ to T_7_ for the injected group (*P* = 0.003). This is a distinct feature of obesity‐associated microbiota due to the ability of methanogenic *Archaea* to enhance the production of short‐chain fatty acids (Samuel and Gordon, 2006; Karlsson *et al.*, 2012). This is consistent with the composition of the calves' gut microbiota treated with florfenicol demonstrating increased in energy harvesting bacteria, resembling microbiota of obese/overweigh animals and humans.

### Emergence of *E. coli* carrying mobile *mcr‐2*, maintenance of *oqxB* genes and resistance to clinical antibiotics in response to florfenicol

Whole metagenome sequencing of samples at day T_o_ and T_7_ resulted in 27 231 751 trimmed sequences for one non‐medicated and 32 598 705 for two medicated animals. Diverse antibiotic resistance genes were identified in faecal samples of both non‐medicated (Table S1) and medicated animals (Tables S2 and S3) with, overall, no significant differences between them, with two exceptions. First, at day seven, the gut microbiota of the two medicated animals contained detectable amounts of the *mcr‐2* gene (Xavier *et al.*, 2016) DNA‐seq reads, phosphoethanolamine‐lipid A transferase MCR‐2 (NCBI Reference: *NG_051171.1*; Tables S2 and S3). The emergence of *mcr‐*2 alleles in response to florfenicol coincides with a 10‐fold increase in the abundance of *E. coli*, and *mcr*‐2 was originally found on the *E. coli* plasmid pKP37‐BE (NCBI Reference: *LT598652.1*. 38). Nested PCR followed by Sanger sequencing of the first 683 nucleotides of the gene confirmed a 100% match to the *mcr*‐2 gene (Fig. S2), starting non‐medicated samples at T_o_ displayed no PCR products. While we were not able to confirm the presence of the pKP37‐BE plasmid in the samples by PCR, it is very likely that the *mcr‐2* allele occurs on this vector due to the complete match of the identified sequence to the reference.

**Figure 5:**
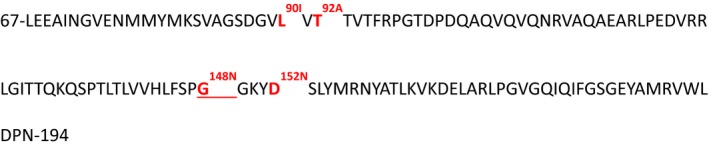
The protein sequence of the metagenomic *OqxB* gene found on the *E. coli* plasmid pOLA52 (EU370913.1:48049‐48434 nucleotide positions). The cloned DNA Sanger sequences were translated into amino acid sequences by using the NCBI ExPASy Translation Tool. The 128 amino acid translated sequence of the N‐terminus (from the 67th to 194th amino acid) is presented. The four different types of amino acid mutations found in the metagenomic sequences are shown in red with superscript letter showing the amino acid substitutions, L90I, T92A, G148N and D152N. The G148N mutation (underlined) was found in all sequenced clones

Second, analysis of the prevalence of quinolone resistance genes revealed that DNA‐seq reads for *oqxB*, a constituent of an RND‐type multidrug efflux pump resistance to olaquindox in *E. coli* (Hansen *et al.*, 2004), were equally present in all tested T_o_ samples. Surprisingly, the *oqxB* gene was detected by neither DNA‐seq nor PCR in the non‐medicated sample at day seven. However, upon the florfenicol treatment, the number of *oqxB* sequences for the two medicated samples at T_o_ and T_7_ was being maintained at a similar level 36 ± 9.5 and 43.7 ± 0.4 respectively (Table S4). It has been reported that the *oqxB* gene occurs on the plasmid pOLA52 (NCBI Reference: *EU370913.1*; Sørensen *et al.*, 2003). Sanger sequencing of the nested PCR fragments of 386 bp (48049 to 48434 nt positions within pOLA52), the sequence which is predicted to be within the drug efflux channel (Sun *et al.*, 2014; Fig. S3), of the metagenomic DNA of medicated samples, confirms the presence of the *oqxB* gene at T_7_. At the DNA level, silent and missense point mutations were found that translated into four sets of mutated polypeptides (Fig. 5). The G148N amino acid substitution was the main feature of all sequenced clones which resulted in appearance of the PN motif. The G148N substitution also occurred on its own, along with L90I or T92A and alongside both L90I and D152N. Hence, polypeptides with either single, double or triple amino acid substitutions were present in the metagenomic *oqxB* gene. Analysis using a combination of molecular modelling and Monte Carlo refinement was used to examine the potential structural consequences associated with substitutions at each locus. It was revealed that the mutations can be divided into two clusters G148N (Fig. S4), D152N (Fig. S5) and L90I/T91A (Fig. S6) and are located in the drug efflux channel and at the interface between OqxA and OqxB respectively. L90I/T91A, despite the apparent physiochemical similarity between Leu and Iso the β‐branched nature of Iso, can influence the secondary structure formation in β‐sheets and α‐helices. *In silico* mutagenesis and Rosetta refinement were used to mimic the mutation (L90I) observed in the cluster. Twenty‐seven of the 30 independent Monte Carlo simulations revealed a break in the β‐sheets and subsequent reorientation of a juxtaposed helical region (P56‐A70). This region of helix is directly exposed to the drug efflux channel and has the potential to alter its shape. Examination of the effect of T91A which is also located in this region revealed in contrast, the *in silico* mutation did not have a marked effect on the secondary structure at this locus. However, the substitution with an Ala at this position does result in the loss of interaction through a hydrogen bond with Ser82 in a juxtaposed β‐sheets and reordering of this area. This also results in the change of position in the helical region P56‐A70 and subsequent reordering resulting in a larger aperture in the efflux channel. G148N/D152N, the location of the second cluster of mutations, sits at the interface between the membrane‐bound OqxB and soluble scaffold protein OqxA of the complex. G148N is located at the interface of 2 β‐sheets. The *in silico* substitution of Asn at this locus results in a further inter β‐sheet interaction with Asp323 located on a juxtaposed β‐sheet. This further hydrogen bond represents an increased stability of approximately 61kcal/mol in this region of secondary structure. For D152N, this mutation is also at the interface between OqxA and OqxB. Visual inspection of the reference sequence reveals that this position (A152) in OqxB is juxtaposed to a charged cluster in an associated chain including an acid residue A281. The substitution of Asn may provide a better complementarity between the subunits which is reflected in the molecular modelling with the two positions becoming closer in the mutant receptor after refinement compared to the reference sequence. The additional interactions in the mutated protein sequence will not only result in local increases in stability but taken together also increase the stability of the complex as a whole. The enhanced thermodynamic stability of the complex would be predicted to lead to an increased protein half‐life and as such increase efflux capacity of the host.

The presence of the pOLA52 plasmid in the treated samples at T_7_ was confirmed by Sanger sequencing of nested PCRs of the metagenomic DNA targeting a randomly selected sequence of 215 bp within the plasmid. These results strongly suggest that preventive antibiotic treatment of healthy animals promotes growth of *E. coli* that carry clinically relevant resistance genes on mobile elements, increasing a risk of the spread of resistance within farm and human populations.

## Discussion

We investigated the impact of a preventive antibiotic (florfenicol) on the gut microbiome and antibiotic resistome of healthy calves. The results show that such therapy has a pleiotropic effect on the gut microbiota composition leading to emergence of an obesogenic and dysanaerobic, disease prone bacterial community that carries clinically relevant mobile antibiotic resistance determinates. First, the selection of obesity‐related microbes without changes in the diet was observed, suggesting that antibiotic treatments may be a key determining factor of obesity. Second, dysbiosis was found during the treatment with an expansion of facultative anaerobes that may trigger inflammation and affect the immune system. Finally, the observed dysbiosis was associated with a significant expansion of *E. coli* carrying clinically important mobile antibiotic resistance genes. This is consistent with a real risk of the spreading of *E. coli* carrying resistance determinants within and between environmental and clinical settings that will diminish the ability to treat bacterial infections with antibiotics. The data strongly argue that the primary outcome of AB therapy is dysbiosis, which in turn propagates obese state bacteria and thus may facilitate the spread of a wide range of resistance genes due to enhancing the aerobic environment of the gut.

The phyla *Firmicutes* and *Bacteroidetes* comprise around 90% of the gut microbiota of the calves. These provide the animals with energy harvested from indigestible and poorly digestible polysaccharides (Eckburg *et al.*, 2005; Ley *et al.*, 2008). Previous analyses of animal and human microbiota have shown that antibiotic treatments can cause transient changes in the composition of the gut microbiota (Sommer and Dantas, 2011). Antibiotics such as tetracyclines, glycopeptides and macrolides administered individually or in combination (e.g. ASP250) have often been used to promote weight gain in animals (Moore *et al.*, 1946; Cromwell, 2002; Looft *et al.*, 2012). Equally, weight gain in humans, both adults and children (Santacruz *et al.*, 2010; Angelakis *et al.*, 2012; Gao *et al.*, 2015), has been observed with some of antibiotics (e.g. tetracycline, macrolides, sulphonamides); however, these effects on health have been disregarded until recently. It is not clear whether the observed antibiotic‐mediated weight gain is a result of improved, infection‐free gut microbiota, the perturbation of its composition or a combination of both. There is however mounting evidence to support the view that changes in the composition of the gut microbiota play a key role in the amount of energy produced from the diet without any changes in nutrient consumption. Significant increases in the abundance of both methanogenic *Archaea* and *Prevotellaceae* spp (which have been observed following antibiotic treatment in this work) are also linked to obesity in humans and animals. *Prevotellaceae* are H_2_‐producing *Bacteroidetes* (Zhang *et al.*, 2009; Everard *et al.*, 2013) which during the fermentation process enable accumulation of H_2,_ reducing the yield of ATP and leading to a gradual decrease in fermentation efficiency. The methanogenic *Archaea* are believed to not only have a role in obesity through promoting caloric intake from otherwise indigestible polysaccharides (Samuel and Gordon, 2006; Turnbaugh *et al.*, 2006; Dridi *et al.*, 2011), but alongside an increase in *Prevotellaceae* can utilize the excess H_2_ to produce acetate, a highly absorbed short‐chain fatty acid (Chakraborti, 2015). Hence, the coexistence of *Prevotellaceae* with methanogenic *Archaea* species in the gut of obese mammals allows for greater efficiency of dietary polysaccharide fermentation and increases their conversion into short‐chain fatty acids, leading to excessive energy storage. It has been suggested that *Bacteria*–*Archaea* syntrophy may be a novel biomarker of susceptibility to obesity (Zhang *et al.*, 2009). In this study, the improved energy harvesting capacity of the calves’ gut microbiota was found to occur without any diet alteration. This is consistent with the idea that an increase in the body fat may occur without any increase in food intake (Bäckhed *et al.*, 2004) and supports the view that the bacterial composition of the gut drives the energy harvesting from the diet.

In the healthy mammalian gut, the hypoxic environment governs the composition of the bacterial community, favouring obligate anaerobes such as *Firmicutes* and *Bacteroidetes*. In turn, they utilize oxygen via β‐oxidation of microbiota‐produced butyrate to carbon dioxide, the main energy production pathway which is critical for the development of the host immune system. Hence, a disruption of anaerobiosis (dysanaerobiosis) due to inflammation (Seksik *et al.*, 2003; Krogius‐Kurikka *et al.*, 2009; Wang *et al.*, 2012; Fei and Zhao, 2013) or treatment of infections with antibiotics, (Bohnhoff *et al.*, 1954; Saito, 1961; Vollaard *et al.*, 1992) for example, leads to an increased oxygenation of the colon and expansion of facultative anaerobic *Proteobacteria* via aerobic respiration. During dysbiosis, energy is also obtained through anaerobic glycolysis, (Donohoe *et al.*, 2012) which does not require oxygen leading to increased epithelial oxygenation (Cabello, 2006). The resultant dysanaerobiosis, an increase of oxygen that disrupts anaerobic environment in the gut, would enable selection for facultative anaerobes, including commensal and pathogenic *E. coli* by aerobic respiration. In this study, florfenicol treatment caused an abnormal escalation of *Proteobacteria* (mainly *E. coli*), resulting in dysbiosis of the gut microbial community which can have a number of consequences as it enhances the risk of disease (Shin *et al.*, 2015; Litvak *et al.*, 2017). *Escherichia spp* continually produce ethanol which increases the permeability of the intestinal wall and promotes colonization by obligate aerobes, characteristics linked to inflammation and obesity. This can lead to certain environmental toxins such as dimethylarsine which can be metabolized by *E. coli* to produce toxic micrometabolites, potential carcinogens of the gut (Yamanaka *et al.*, 1989). Increased oxygen availability which promotes an expansion of *E. coli* can also pave the way for growth of aerobic pathogens, hence offsetting the benefit of using preventive AB therapies. Furthermore, *E. coli* are also a potential human pathogen that can carry antibiotic resistance determinants on mobile elements, raising a possibility of spreading resistance within and between communities.

Taken together, an antibiotic‐induced dysbiotic expansion of facultative anaerobic *E. coli* can predispose the host to disease, such as intestinal inflammation and cancer. It also can drive an *Enterobacteriaceae* enteric pathogen colonization which carries clinically relevant transmissible antibacterial resistances. Therefore, practices of a preventive antibiotic treatment of healthy animals must consider the potential threat to animal health as well as a high risk of zoonotic transfer of pathogens carrying mobile resistances to the human population.

Antibiotic therapy in livestock plays a key role in the selection of resistant bacteria (Witte, 1998) that can be transmitted between animals, humans and the environment (Cabello, 2006). Recently, the *mcr*‐1 allele conferring a novel plasmid‐borne colistin resistance gene was discovered in food animals (Liu *et al.*, 2016) and it has now been detected across all continents (Acrilla *et al.*, 2016; Hu *et al.*, 2016; Olaitan *et al.*, 2016; Webb *et al.*, 2016). The *mcr*‐1 resistance gene is found on a highly transmissible plasmid, namely IncX_4_ which is a narrow host range mobile element of *Enterobacteriaceae* with a lack of fitness burden on bacterial host (Liu *et al.*, 2016). Recent evidence strongly suggests transfer of *mcr*‐1 from animals to humans while it also can be spread in hospitals without colistin pressure (Paterson and Harris, 2016; Al‐Tawfiq *et al.*, 2017; references therein Sun *et al.*, 2018). Subsequently, *mcr*‐2, another plasmid‐mediated colistin resistance gene, has been identified in porcine and bovine colistin‐resistant *E. coli* (Xavier *et al.*, 2016). Like *mcr*‐1, *mcr*‐2 is a phosphoethanolamine transferase which also occurs on an IncX_4_ plasmid (pKP37‐BE) that makes this resistance highly transmissible. While we have not been able to confirm the presence of pKP37‐BE in the samples where PCR clones containing the *mcr*‐2 sequences were identified (probably due to still low abundance of the DNA), it is very likely that *mcr*‐2 found in this study is also occurring on pKP37‐BE or another IncX_4_ plasmid. This is the first case of the emergence of the *mcr*‐2 allele in the UK and in livestock in response to antibiotic treatment. Importantly, cows at the farm descended from stock purchased from Belgium where the first emergence of the *mcr‐2* allele was discovered (Xavier *et al.*, 2016). The finding of this work that 10‐month‐old calves (the first generation which has not been subjected to antibiotic pressure before) microbiota possess *mcr*‐2, which is likely to have originated in Belgium, this implies that the resistance allele has come vertically from their mother and that *mcr*‐2 is as stable as *mcr*‐1 due to the allele being present without AB pressure (Hu *et al.*, 2016). However, *mcr*‐2 transmission via an environmental route within the farm cannot be ruled out. This strongly argues that antibiotic resistance determinants can also be spread due to international livestock trading. Therefore, this finding strongly supports a view that screening for stable antibiotic resistances in agriculture, such as *mcr* alleles (Xavier *et al.*, 2016), should be included in epidemiological surveillance to monitor and control the dissemination of animal‐borne resistance which is of clinical relevance in humans.

Efflux pumps are associated with resistance to multiple antimicrobial agents due to their ability to efficiently extrude a broad range of chemicals from the bacterial cell, thereby lowering the intracellular level of antibiotics. This mechanism of resistance is seen as the first line of defence which plays a key role in the selection of novel resistance genes. This gives the cell a time to develop a targeted (multi)drug resistance to higher concentrations of the antibiotic and/or drives the accumulation of additional gain‐of‐resistance mechanisms. Moreover, efflux pump genes can occur on mobile elements that can be transmitted to pathogenic bacteria (Louw *et al.*, 2009; Martinez, 2009; Machado *et al.*, 2012; Schmalstieg *et al.*, 2012; Gumbo, 2013). The cloned and sequenced region of 312 nts, from 271 to 583 nts, of the metagenomic *oqxB* gene is a part of the predicted efflux pump domain. Based on the crystal structure of the *E. coli* AcrB protein, the *E. coli* chromosomal efflux system, AcrAB‐TolC (Murakami *et al.*, 2002), we have developed a model of the OqxA/B drug efflux transporter. The V89‐N194 sequenced region of the *oqxB* spans two regions of the protein which are critically involved with drug recognition/removal and intra/inter‐subunit stability. The positions Leu90 and Thr91 are located in the RGD region of the protein which is responsible for substrate recognition and ultimately antibiotic removal from the host. Mutations in this region may alter the poly‐substrate specificity of the transporter but based on the increase diameter of the efflux channel a higher rate of antibiotic removal may be predicted. In addition to this, further mutations at positions Gly148 and Asp152 in OqxB result in enhanced thermodynamic and kinetic stability of the complex as a whole through additional intra‐ and inter‐molecular interactions. Collectively, these mutations would give rise to an efflux pump which is predicted to have both a longer half‐life and also an enhanced ability to remove drugs from the host. The *oqxAB* operon carries a resistance‐nodulation‐cell division‐type efflux pump that confers resistance to the growth promoter olaquindox as well as to ampicillin and chloramphenicol (Sørensen *et al.*, 2003). In this study, the identified *oqxB* gene of the *oqxAB*‐encoded pump resistance determinant occurs on an IncX_1_ plasmid, pOLA52 which is also highly mobile due to the presence of an IS26 element and a T3 transposon (Sørensen *et al.*, 2008). Alarmingly, *oqxAB*‐positive commensal *E. coli* isolates have been found among farmworkers who have undergone no previous antibiotic treatment or been admitted to the hospital, suggesting horizontal transfer of the *oqxAB* resistance (van den Bogaard *et al.*, 2001; Zhao *et al.*, 2010). Direct transmission of *oqxAB*‐positive *E. coli* between humans and livestock has also been reported (Moodley and Guardabassi, 2009). Thus, maintenance of *E. coli* carrying *oqxAB* in animals in response to florfenicol and possibly to other antibiotics may enhance its spread to humans. This, in turn, may increase the risk of horizontal transfer of resistance to human pathogens.

## Implications

Florfenicol treatment of calves resulted in changes in the gut microbiota, so that it resembles the one of obese humans/animals without any diet alteration. Obesity is a major public and clinical health concern affecting around 400 million people globally. While diet contributes to the diversity of the gut microbiota (Bäckhed *et al.*, 2005) and affects body mass, however, it is shown that this increase may occur without changes in food consumption (Ley *et al.*, 2006). Hence, the improved energy harvesting capacity of the healthy gut microbiota in response to the antibiotic may contribute to enhanced energy intake while maintaining the same food intake. Therefore, its potential impact on obesity is to be taken into consideration by health professionals during the treatment of clinical infections.

As a result of dysbiosis, a significant enrichment in the prevalence of facultative anaerobes such as *E. coli* in response to a preventive antibiotic treatment increases the risk of aerobic pathogen colonization in the healthy host gut and predisposition to a number of diseases. Additionally, dysbiosis changes permeability of the colon and decreases the defence mechanisms of the host against bacteria‐driven toxins. Therefore, a positive outcome of antibiotic preventive therapies, namely protecting the host against potential infection, may inadvertently lead to a number of severe side‐effects. Of clinical concern, the observed expansion of *E. coli* coincides with the emergence and preservation of *E. coli* mobile‐mediated resistance determinants, a colistin resistance allele *mcr*‐2 and a member of the multidrug efflux pump *oqxAB* operon, the *oqxB* gene, respectively (Hansen *et al.*, 2004; Xavier *et al.*, 2016). These genes confer resistance to antibiotics of first choice (e.g. quinolones) and last resort (e.g. colistin) to treat multidrug‐resistant hospital bacterial infections. The emergence of the novel *mcr‐2* gene on Belgian commercial farms where the AB therapy is strictly regulated (Xavier *et al.*, 2016) showed primary evidence of *E. coli* carrying mobile elements with stable resistances, such as the *mcr* allele (Shen *et al.*, 2016) to preserve genes which might confer the host bacteria novel adaptive properties, for example ability for stable colonization in strictly anaerobic environment and/or survive in response to AB pressure. The presence of *mcr*‐2 in the gut of animals on a small rural farm where non‐colistin prophylactic antibiotic therapy is commonly used is consistent with the previous findings and its emergence coincides with expansion of *E. coli* carrying this resistance suggesting that *mcr* alleles are important for adaptive responses. *E. coli* carrying efflux pump resistance determinants, such as *oqxAB,* can be less susceptible to a broad range of antibiotics due to acquiring new mutations, hence enhancing their surviving properties. Furthermore, the recently discovered plasmid‐born *mcr‐1* already is found on extended‐spectrum β‐lactamase multidrug resistance plasmids (e.g. Li *et al.*, 2018), while plasmid‐mediated multidrug efflux cassettes are also linked to multidrug‐resistant infections in the hospital setting (Kim *et al.*, 2009). While it is shown that originally these resistance elements have been selected due to a direct effect of polymyxin and olaquindox to promote animal growth their enrichment in the animal gut in response to florfenicol, a non‐cognate class of antibiotic was alarming. Nevertheless, this represents a collateral effect of antibiotic therapies that may lead to an increase in resistance to non‐administered drugs (Looft *et al.*, 2012) driving the development and spread of a wide range of clinical resistance regardless of the specificity of antibiotics; mobilized colistin resistance determinants have already been identified in clinical settings. Identification of the plasmid‐mediated *mcr*‐2 and *oqxB* resistances marks a paradigm shift in our understanding of the spread and development of resistance as until recently they were sequestered to the chromosome (hence, were less likely to be associated with multidrug‐resistant plasmid‐mediated infections which in turn were relatively easy to treat with drugs that target the chromosomal genes) and capable of vertical transmission only. Hence, routine screening for mobile resistance genes in livestock must be considered as a prerequisite for any farm using antibiotics as a preventative therapy on food animals in order to contain the spread of plasmid‐borne resistance determinants. Based on our results, we would argue that the short‐term benefit of a given preventive antibiotic therapy must be carefully assessed and weighed against the collateral effects on promoting antibiotic resistance and that novel screening policies for clinically important bacteria occurring in agricultural environments are urgently required.

## Experimental procedures

### Samples

In this study, the 3Rs principles have been applied in order to reduce the number of animals used. Six seven‐month‐old calves were divided into two groups of three. One group (‘medicated’) received an injection of Nuflor^®^ (containing florfenicol, a fluorinated synthetic analogue of thiamphenicol) which is commonly used to prevent respiratory tract infections in cattle (e.g. *Mannheimia haemolytica*, *Histophilus somni* and *Pasteurella multocida*), 40 mg/kg body weight, while the other group (‘non‐medicated’) received no antibiotics. The antibiotic was subcutaneously injected by the college veterinarian as per manufactory recommendation (MSD Animal Health).The calves were housed in decontaminated and highly controlled setting at the Moreton Morrell College Farm, Warwickshire College. The Warwickshire College Board approved the use of college animals for this study. Animals were treated under the supervision of the registered farm veterinarian and in accordance with the Moreton Morrell College Farm guidelines based on the revised Animals (Scientific Procedures) Act 1986 in the UK and Directive 2010/63/EU in Europe. All animals were fed the same diet (*ad libitum* hay and silage and 1 kg of concentrates/animal/day) throughout the study and were not exposed to any antibiotic treatment prior to the study. A week before the study, the medicated and non‐medicated calves were segregated into two separate rooms to prevent cross‐contamination. The cows were observed by the college veterinarian during the period of the study, and no changes in animal health have been found after 7 days of the study. Detail of sample handling is described in Appendix S1.

### Genomic DNA isolation and sequencing

Total genomic DNA (gDNA) was extracted using the QIAamp^®^ DNA Stool kit (QIAGEN^®^). 0.2 g of cell pellet was re‐suspended in 180 μl of 1 × PBS and processed according to the manufacturer’s instructions. The concentration of purified gDNA was measured by spectrophotometry at A_260_. Total DNA was used for whole (meta)genome shotgun sequencing and as template for the amplification and sequencing of the V3‐V4 hypervariable regions of the bacterial 16S small subunit ribosomal RNA gene for metagenomic profiling. Details of the sequencing protocols, including primer sequences, are provided in Appendix S1. All sequence data were submitted to the European Nucleotide Archive (ENA), under the study accessions PRJEB33144 (for the 16S rRNA data) and PRJEB33145 (for the whole metagenome shotgun sequence data).

### Metagenomic profiling of bacterial communities

For each sample, 2 × 300 bp sequencing read pairs from 16S amplicons were aligned to each other using flash v1.2.11 (Magoč and Salzberg, 2011). Resulting sequences were processed to identify and remove amplification primer sequences from their 5′ and 3′ ends, retaining only those with an identified amplification primer sequence at each end, using cutadapt v1.9.1 (Martin, 2011). Processed sequences containing undefined nucleotides (N's) and those of unexpected size (<350 bp or >450 bp) were removed. To balance sample sizes for each time‐course, read sets were randomly down‐sampled to match the size of the smallest of the three libraries prior to clustering. In each size‐matched read set, sequences were de‐replicated by clustering identical sequences and clusters of size 1 (likely to be enriched for erroneous sequences) were removed. The remaining filtered, de‐replicated reads were clustered at 97 % sequence identity. De‐replication and clustering were performed using vsearch v1.1.1 (Rognes *et al.*, 2016). Clusters were further filtered to remove putative chimeric sequences using the ‘uchime denovo’ option in vsearch. The remaining, putatively non‐chimeric, clusters were given a taxonomic assignment using SINA (Pruesse *et al.*, 2012) using default parameters, the ‘search and classify’ option and the SILVA database as the primary source of taxon labels. In each case, results from SINA were processed by trimming taxonomic labels to the required rank and summing the read numbers for clusters with identical labels.

### Profiling the bacterial community ‘resistome’ using whole‐genome metagenomic shotgun sequencing

To assay the ‘resistome’ in each sample, sets of resistance‐associated genes were obtained from Resfinder (https://cge.cbs.dtu.dk/services/ResFinder/) and used to make a set of databases suitable for use with BLAT (Kent, 2002). Reads were processed by removing sequencing library adapter sequences using cutadapt v1.9.1 (Martin, 2011), retaining only reads longer than 50 bp and containing no ‘N’s after trimming. Trimmed reads were converted to FASTA format and ‘de‐replicated’ (identical reads collapsed into one read), using vsearch v1.1.1 (Pruesse *et al.*, 2012). This was done to (i) make later analysis more computationally efficient and (ii) enable artefacts such as PCR duplicates to be accounted for in later analyses. De‐replicated reads from each sample were searched against each gene set using BLAT (Kent, 2002). All reads and target genes were translated in all six reading frames and aligned as proteins in order to capture reads from species more distantly related from the ‘reference’ sequences. Reads matching ‘reference’ sequences in each ‘database’ were quantified as the number of unique sequence matches (i.e. de‐replicated reads) and the total number of matches (i.e. including multiple identical reads) and expressed as counts and as proportions of the total library size.

### Semi‐quantitative PCR amplification of 16S rRNA and *uidA* genes

A significant increase in the DNA‐seq reads for *Escherichia* spp. at T_7_ was validated by semi‐quantitative PCR for the *uidA* gene and using the 16S rRNA gene as a normalization control. PCR conditions are presented in Appendix S1.

### PCR, cloning and Sanger sequencing of the *mcr‐2* and *oqxB* genes

To confirm the DNA‐seq data with regard to the emergence of the *mcr‐2* gene and presence or absence of *oqxB* visualization of nested PCR bands followed by cloning and Sanger sequencing was carried out. Details of cloning approaches are described in Appendix S1.

### Molecular modelling

Molecular models of OqxA and OqxB were generated using the crystal structure of AcrAB‐TolC Multidrug Efflux Pump (PDB http://www.rcsb.org/pdb/search/structidSearch.do?structureId=5V5S.pdb) using Modeller (Sali and Blundell, 1993). Hundred models were generated and initially ranked using OPUS_PSP (Lu *et al.*, 2008). Five top scoring models were refined using the membrane protein module from Rosetta (Alford *et al.*, 2015). Point mutations were made to the wild‐type sequence using Rosetta. Changes in secondary structure and relative motions of secondary structure elements were analysed using an in‐house python script.

### Statistical analysis

rDNA‐seq reads were normalized between samples to total number of read of the samples and presented as percentage of total reads. In order to examine any differences in bacterial compositions over time and between treatment and control groups, a series of 2 (injected vs control, non‐medicated) × 3 (time points) way repeated measures ANOVAs (SPSS v.24) were conducted, and Benjamini–Hochberg test, the false discovery rate (the critical value of <0.033) method, was used (Glickman *et al.*, 2014). Where any significant differences were evident, post hoc pairwise comparisons were employed to determine where the differences lay and where appropriate standard error at 95% confidence interval was used.

## Conflict of interest

The authors declare no conflict of interest.

## Author contribution

L.A., J.R. and I.Y.M. designed research; D.A.D., M.T.F.L., S.C. and I.Y.M. performed research; M.D. and I.Y.M performed statistical analysis; D.A.D., M.T.F.L., G.D.W., J.S., L.A. and I.Y.M. analysed data; and M.T.F.L., G.D.W, J.R. and I.Y.M. wrote the paper.

## Supporting information


**Fig. S1.** Assessment of an increase in the *E. coli* level in response to Nuflor treatment. A series of dilutions of the same sets of gDNAs (240 ng, 120 ng or 60 ng) at To and T7 for the 16S RNA‐ and uidA genes were used for the semi‐quantitative PCR amplification. 1 and 3, a 573 nts PCR fragment of uidA at T7 and To, correspondently; 2 and 4, a 500 nts PCR fragment of the 16S RNA gene at T7 and To, respectively. From this analysis it was apparent that the To uidA sample produced a signal <2% of the Nuflor treated sample at day 7.
**Fig. S2** **. **Sanger sequencing of the nested PCR fragments for first 683 nucleotides of the *mc*r‐2 gene confirmed the DNA‐seq data for the emergence of the mcr‐2 allele.
**Fig. S3** **. **A molecular model of the OqxA/B efflux pump as shown parallel to the membrane.
**Fig. S4** **. **The G148N mutation in OqxB.
**Fig. S5** **. **The D152N mutation in OqxB.
**Fig. S6** **. **The effect of mutations L90I and T92A on the efflux channel.
**Table S1** **. **Change in the number/proportion of matches over the time course for ‘non‐medicated’.
**Table S2** **. **Change in the number/proportion of matches over the time course for ‘medicated 1’.
**Table S3** **. **Change in the number/proportion of matches over the time course for ‘medicated 2’.
**Table S4** **. **Changes in the DNA‐seq *OqxB* reads in response to Nuflor over the time course.

**Appendix S1** **.** Experimental procedures.
