# Supplementary material for: Preventive antibiotic treatment of calves: emergence of dysbiosis causing propagation of obese state‐associated and mobile multidrug resistance‐carrying bacteria
Source: Microb Biotechnol. 2019 Oct 30;13(3):669–82. doi: 10.1111/1751-7915.13496 (PMC7111097; doi:10.1111/1751-7915.13496)
Supplement: Supplementary file 1 — Fig. S1. Assessment of an increase in the E. coli level in response to Nuflor treatment. A series of dilutions of the same sets of gDNAs (240 ng, 120 ng or 60 ng) at To and T7 for the 16S RNA‐ and uidA genes were used for the semi‐quantitative PCR amplification. 1 and 3, a 573 nts PCR fragment of uidA at T7 and To, correspondently; 2 and 4, a 500 nts PCR fragment of the 16S RNA gene at T7 and To, respectively. From this analysis it was apparent that the To uidA sample produced a signal <2% of the Nuflor treated sample at day 7. Fig. S2 . Sanger sequencing of the nested PCR fragments for first 683 nucleotides of the mcr‐2 gene confirmed the DNA‐seq data for the emergence of the mcr‐2 allele. Fig. S3 . A molecular model of the OqxA/B efflux pump as shown parallel to the membrane. Fig. S4 . The G148N mutation in OqxB. Fig. S5 . The D152N mutation in OqxB. Fig. S6 . The effect of mutations L90I and T92A on the efflux channel. Table S1 . Change in the number/proportion of matches over the time course for ‘non‐medicated’. Table S2 . Change in the number/proportion of matches over the time course for ‘medicated 1’. Table S3 . Change in the number/proportion of matches over the time course for ‘medicated 2’. Table S4 . Changes in the DNA‐seq OqxB reads in response to Nuflor over the time course. [file MBT2-13-669-s001.docx]

500 nts

1 2 3 4

**Fig. S1.**

| **Antibiotic Family** | **Unique** |  | **Non-unique** |  |
| --- | --- | --- | --- | --- |
|  | T_o_ | T_7_ | T_o_ | T_7_ |
|  | n (p)^1^ | n (p)^1^ | n (p)^2^ | n (p)^2^ |
| **Aminoglycoside** | 68 (5.4339) | 395 (26.8383) | 71 (5.1108) | 458 (28.5953) |
| **β-lactam** | 61 (4.8745) | 71 (4.8241) | 62 (4.4630) | 73  (4.5578) |
| **Colistin** | 0 (0.0000) | 0 (0.0000) | 0 (0.0000) | 0 (0.0000) |
| **Fosfomycin** | 2 (0.1598) | 1 (0.0679) | 2 (0.1440) | 1 (0.0624) |
| **Fusidicacid** | 0 (0.0000) | 0 (0.0000) | 0 (0.0000) | 0 (0.0000) |
| **Glycopeptide** | 583 (46.5879) | 696 (47.2898) | 634 (45.6375) | 757 (47.2634) |
| **Macrolide** | 702 (56.0973) | 856 (58.1610) | 767 (55.2112) | 1013 (63.2469) |
| **Nitroimidazole** | 28 (2.2375) | 42 (2.8537) | 29 (2.0875) | 49  (3.0593) |
| **Oxazolidinone** | 27 (2.1576) | 58 (3.9408) | 27 (1.9435) | 150 (9.3653) |
| **Phenicol** | 27 (2.1576) | 68 (4.6203) | 27 (1.9436) | 157 (9.8023) |
| **Quinolone** | 36 (2.8768) | 46 (3.1255) | 41 (2.9513) | 48  (2.9969) |
| **Rifampicin** | 0 (0.0000) | 0 (0.0000) | 0 (0.0000) | 0 (0.0000) |
| **Sulphonamide** | 2 (0.1598) | 0 (0.0000) | 2 (0.1440) | 0 (0.0000) |
| **Tetracycline** | 1739 (138.9646) | 1687 (114.6233) | 2395 (172.4002) | 2189 (136.6707) |
| **Trimethoprim** | 6 (0.4795) | 6 (0.4077) | 6 (0.4319) | 7 (0.4370) |

**Table S1**. Change in the number/proportion of matches over the time course for ‘non-medicated’.

| **Antibiotic Family** | **Unique** |  | **Non-unique** |  |
| --- | --- | --- | --- | --- |
|  | T_o_ | T_7_ | T_o_ | T_7_ |
|  | n (p)^1^ | n (p)^1^ | n (p)^2^ | n (p)^2^ |
| **Aminoglycoside** | 52 (6.6760) | 343 (36.0264) | 52 (6.5418) | 362 (36.3362) |
| **β-lactam** | 181 (23.2377) | 94 (9.8731) | 187 (23.5254) | 97 (9.7365) |
| **Colistin** | 0 (0.0000) | 3 (0.3151) | 0 (0.0000) | 3 (0.3011) |
| **Fosfomycin** | 0 (0.0000) | 1 (0.1050) | 0 (0.0000) | 1 (0.1004) |
| **Fusidicacid** | 0 (0.0000) | 0 (0.0000) | 0 (0.0000) | 0 (0.0000) |
| **Glycopeptide** | 325 (41.7252) | 397 (41.6982) | 333 (41.8928) | 406 (40.7528) |
| **Macrolide** | 434 (55.7191) | 574 (60.2891) | 445 (55.9828) | 614 (61.6310) |
| **Nitroimidazole** | 9  (1.1555) | 16 (1.6805) | 9  (1.1322) | 17 (1.7064) |
| **Oxazolidinone** | 49 (6.2909) | 51 (5.3567) | 50 (6.2902) | 69 (6.9260) |
| **Phenicol** | 16 (2.0542) | 48 (5.0416) | 16 (2.0129) | 67 (6.7252) |
| **Quinolone** | 49 (6.2909) | 55 (5.7768) | 50 (6.2902) | 57 (5.7214) |
| **Rifampicin** | 0 (0.0000) | 0 (0.0000) | 0 (0.0000) | 0 (0.0000) |
| **Sulphonamide** | 0 (0.0000) | 1 (0.1050) | 0 (0.0000) | 1 (0.1004) |
| **Tetracycline** | 679 (87.1735) | 784 (82.3460) | 706 (88.8177) | 827 (83.0111) |
| **Trimethoprim** | 3 (0.3852) | 0 (0.0000) | 3 (0.3774) | 0 (0.0000) |

**Table S2**. Change in the number/proportion of matches over the time course for ‘medicated 1’.

| **Antibiotic Family** | **Unique** |  | **Non-unique** |  |
| --- | --- | --- | --- | --- |
|  | T_o_ | T_7_ | T_o_ | T_7_ |
|  | n (p)^1^ | n (p)^1^ | n (p)^2^ | n (p)^2^ |
| **Aminoglycoside** | 40 (5.5312) | 309 (38.3512) | 42 (5.5823) | 338 (40.2849) |
| **β-lactam** | 50 (6.9140) | 78 (9.6809) | 50 (6.6456) | 79 (9.4157) |
| **Colistin** | 0 (0.0000) | 5 (0.6206) | 0 (0.0000) | 5 (0.5959) |
| **Fosfomycin** | 0 (0.0000) | 0 (0.0000) | 0 (0.0000) | 0 (0.0000) |
| **Fusidicacid** | 0 (0.0000) | 0 (0.0000) | 0 (0.0000) | 0 (0.0000) |
| **Glycopeptide** | 343 (47.4300) | 395 (49.0249) | 351 (46.6524) | 405 (48.2703) |
| **Macrolide** | 380 (52.5463) | 479 (59.4505) | 408 (54.2285) | 517 (61.6191) |
| **Nitroimidazole** | 14 (1.9359) | 19 (2.3582) | 14 (1.8608) | 19 (2.2645) |
| **Oxazolidinone** | 20 (2.7656) | 55 (6.8263) | 22 (2.9241) | 69 (8.2238) |
| **Phenicol** | 30 (4.1484) | 46 (5.7092) | 30 (3.9874) | 61 (7.2703) |
| **Quinolone** | 26 (3.5953) | 49 (6.0816) | 26 (3.4557) | 50 (5.9593) |
| **Rifampicin** | 0 (0.0000) | 0 (0.0000) | 0 (0.0000) | 0 (0.0000) |
| **Sulphonamide** | 0 (0.0000) | 2 (0.2482) | 0 (0.0000) | 2 (0.2384) |
| **Tetracycline** | 722 (99.8380) | 730 (90.6030) | 749 (99.5518) | 768 (91.5348) |
| **Trimethoprim** | 7 (0.9680) | 3 (0.3723) | 7 (0.9304) | 4 (0.4767) |

**Table S3**. Change in the number/proportion of matches over the time course for ‘medicated 2’.

| Sample | T_o_ | T_3_ | T_7_ |
| --- | --- | --- | --- |
| s63, Injected | 35 | 24 | 13 |
| s66, Injected | 49 | 20 | 43 |
| s76, Injected | 26 | 30 | 44 |
| s77, Control | 34 | 0 | 0 |

**Table S4**. Changes in the DNA-seq *OqxB* reads in response to Nuflor over the time course.


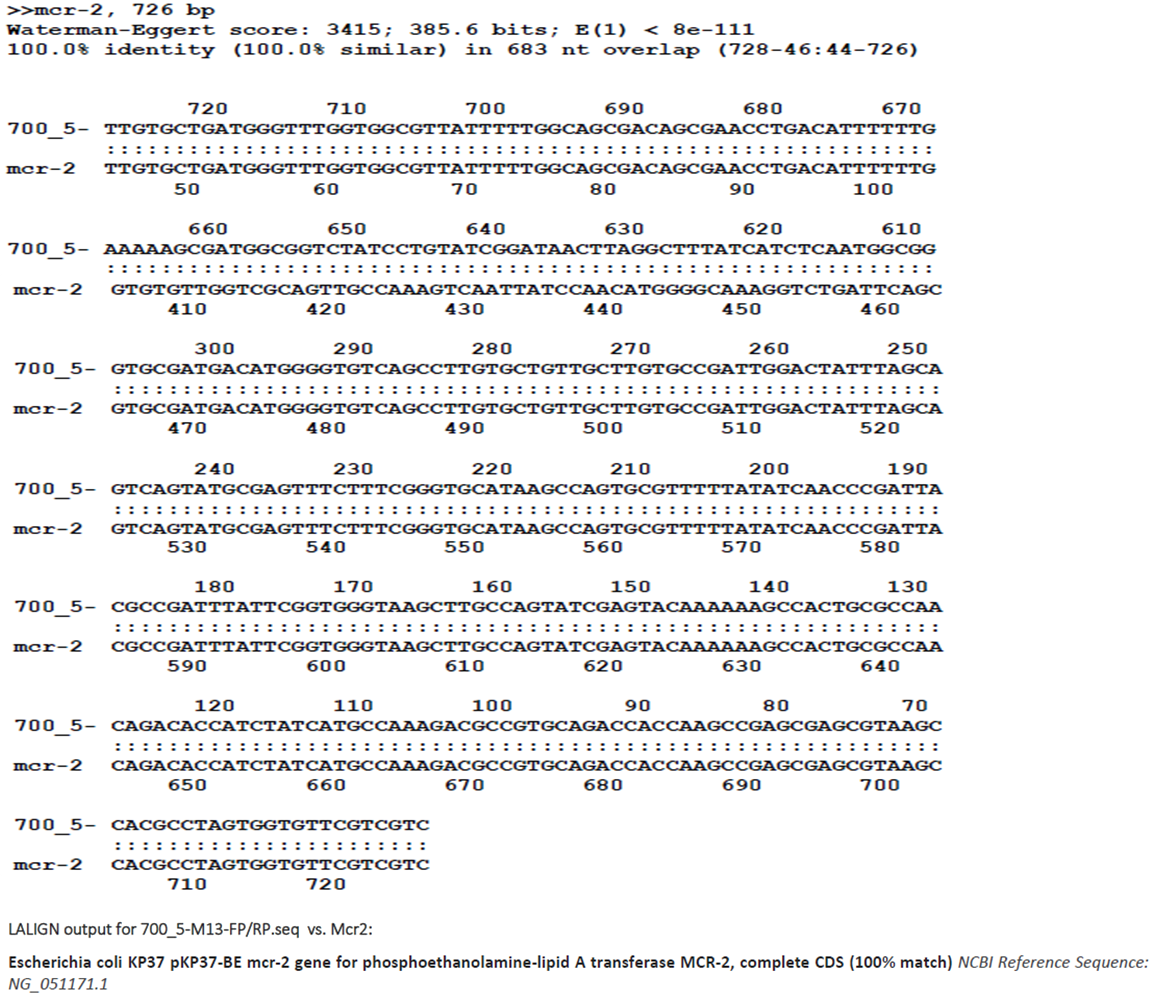


**Fig. S2.** Sanger sequencing of the nested PCR fragments for first 683 nucleotides of the *mc*r-2 gene confirmed the DNA-seq data for the emergence of the mcr-2 allele.

**A**


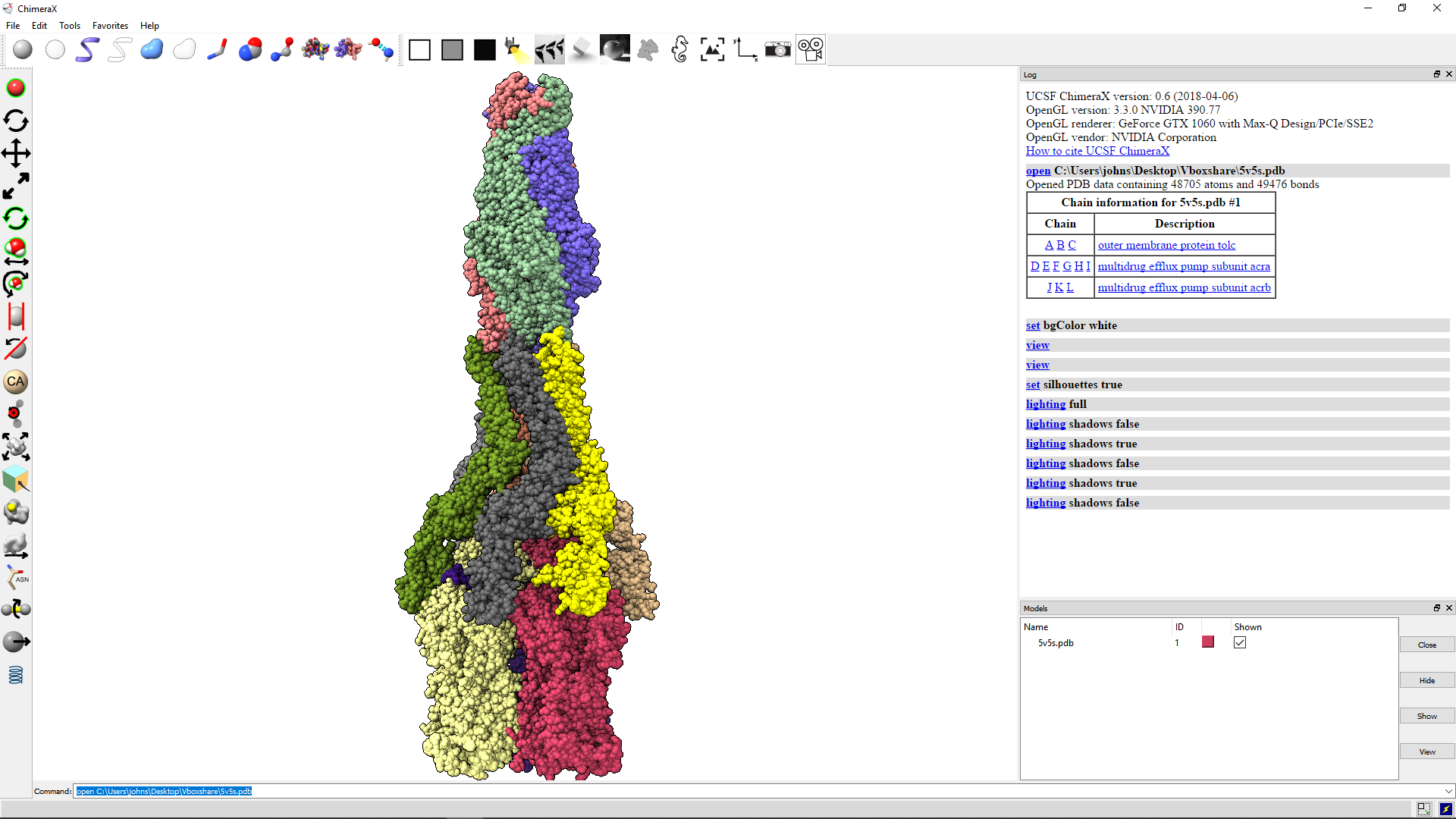


OqxB

OqxA

TolC

TolC, Subunit A

OqxA, Subunit A

**B**


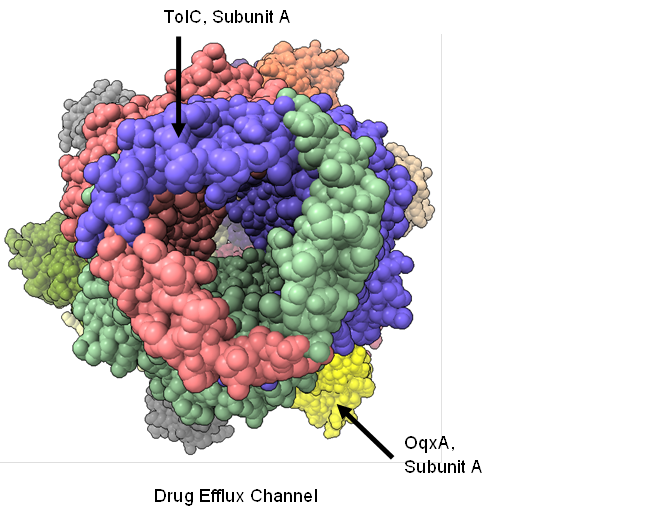


**Fig. S3.** **(A)** A molecular model of the OqxA/B efflux pump as shown parallel to the membrane.

**A**

OqxB

OqxA

TolC


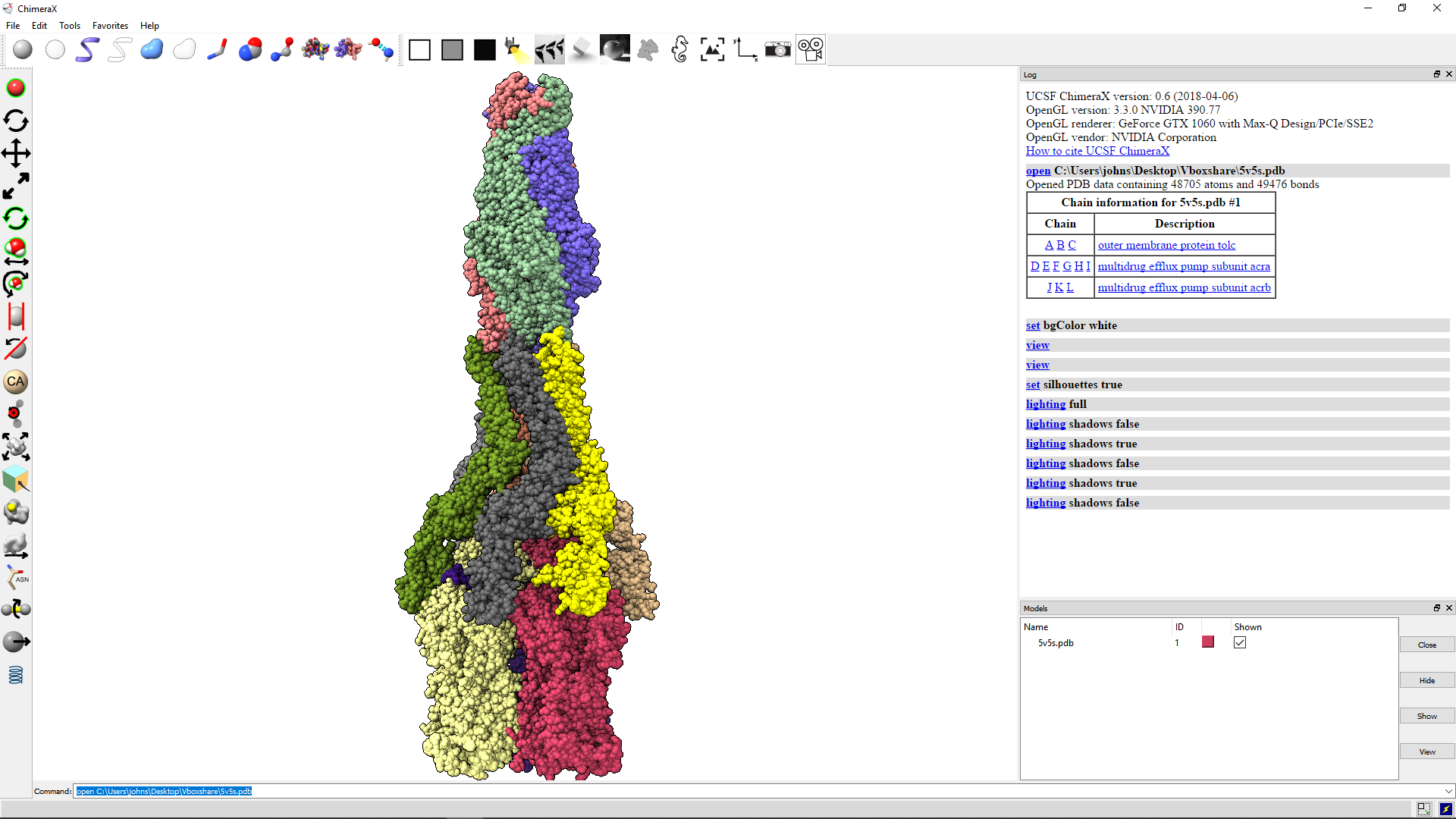


**B**


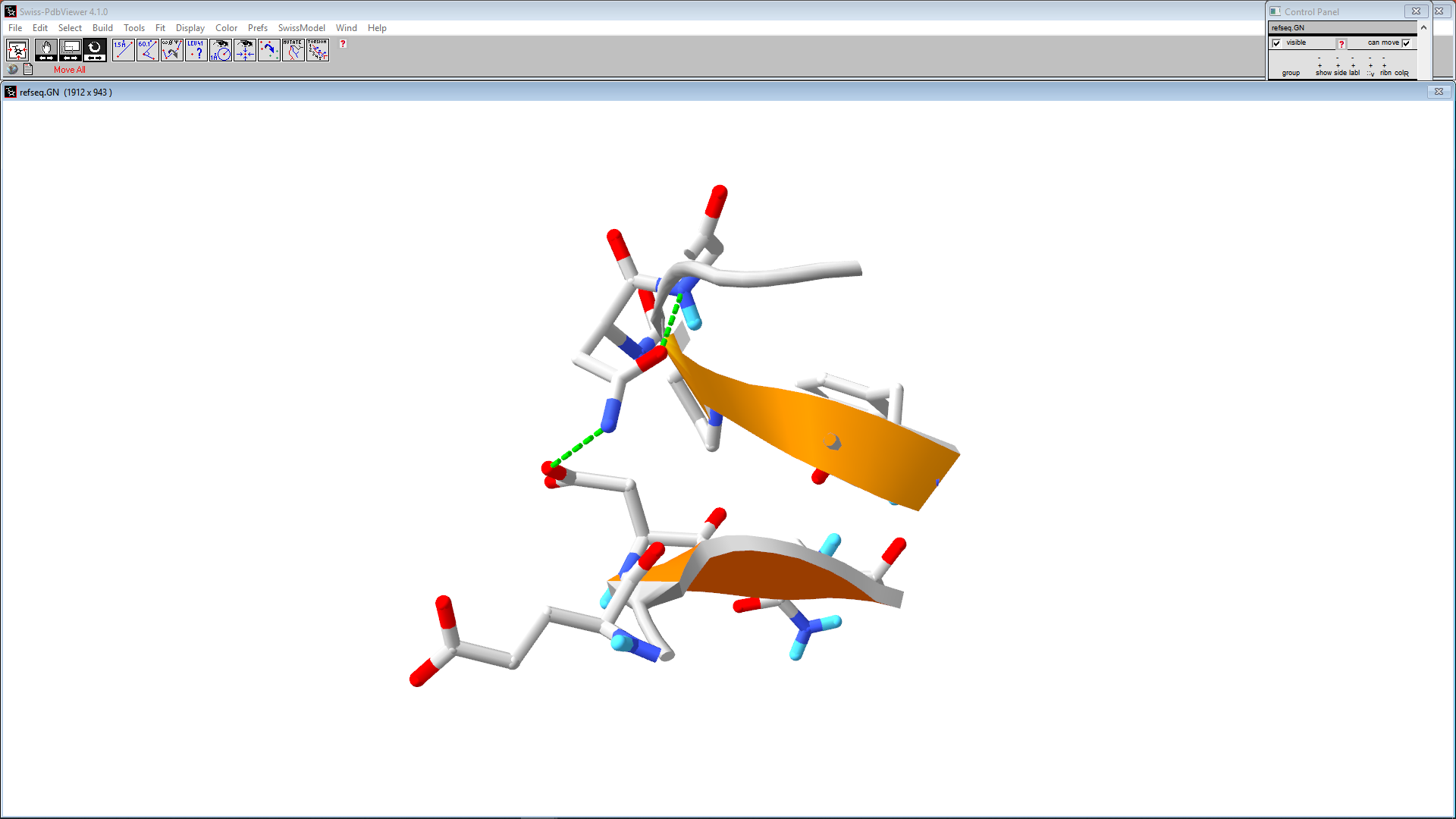


G148N

Asp^323^

**Fig. S4**. The G148N mutation in OqxB.

**A**


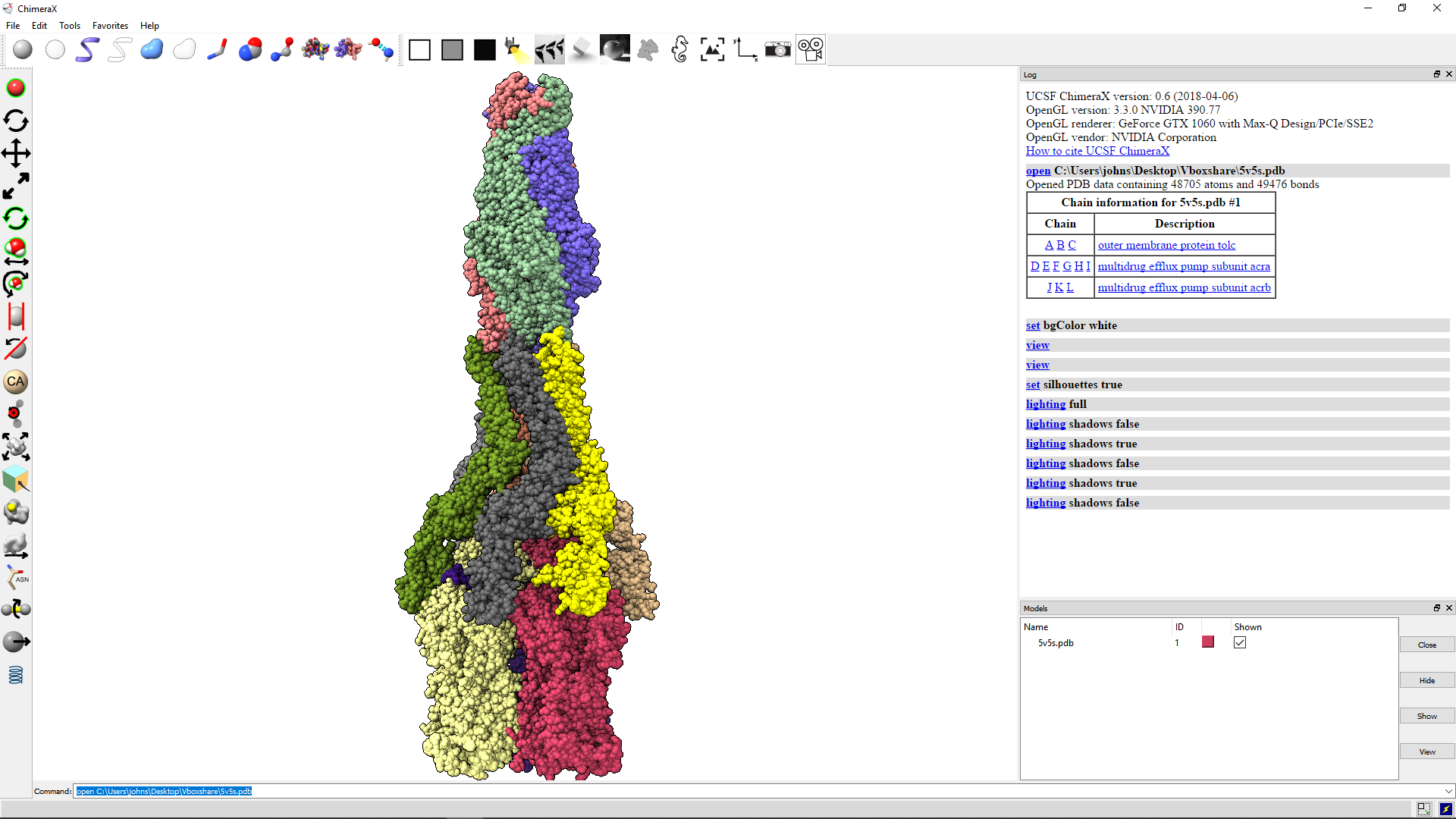


OqxB

OqxA

TolC

**B**


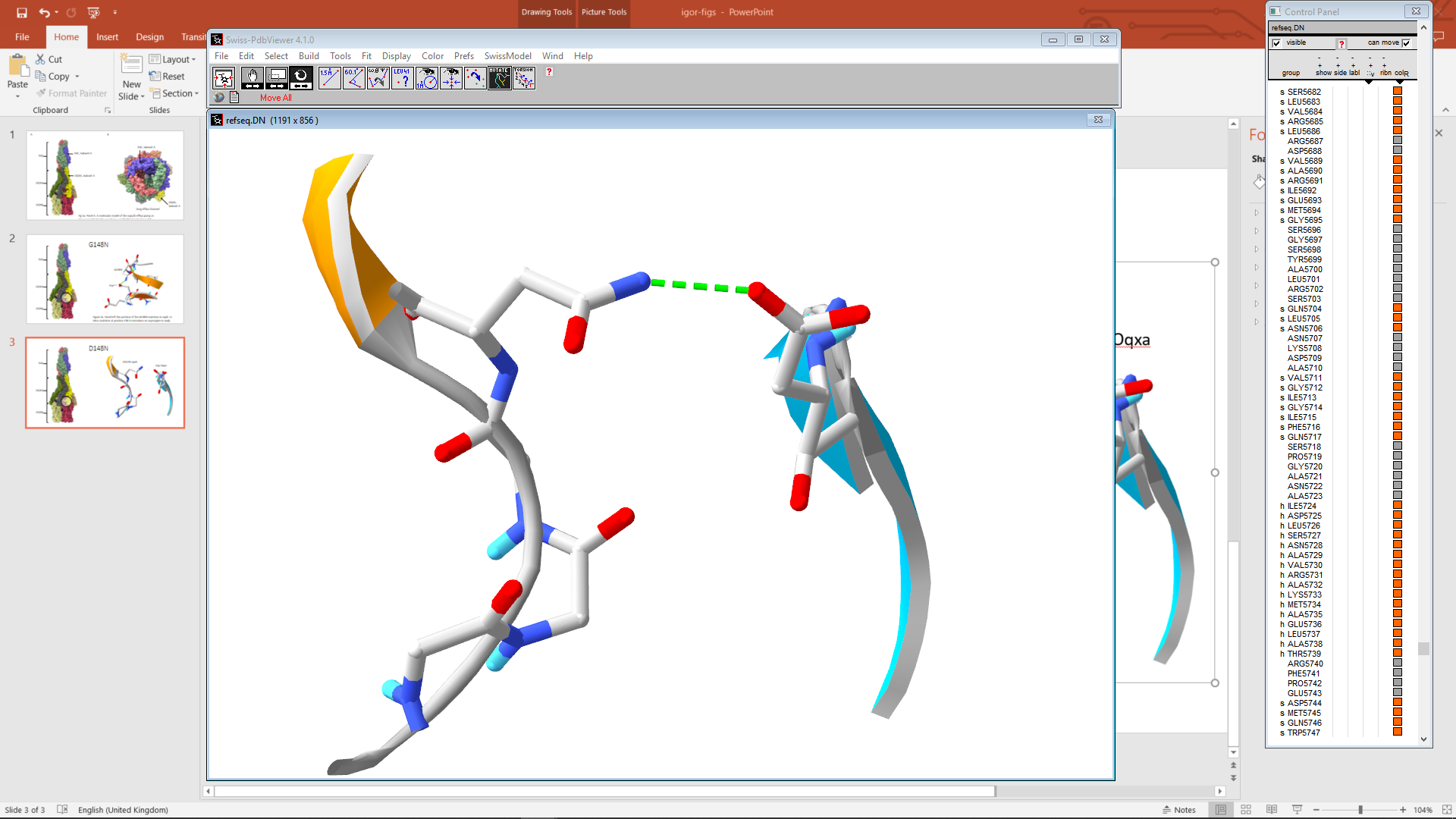


Asp^281^ OqxA

D152N OqxB

**Fig. S5**. The D152N mutation in OqxB.

**A**


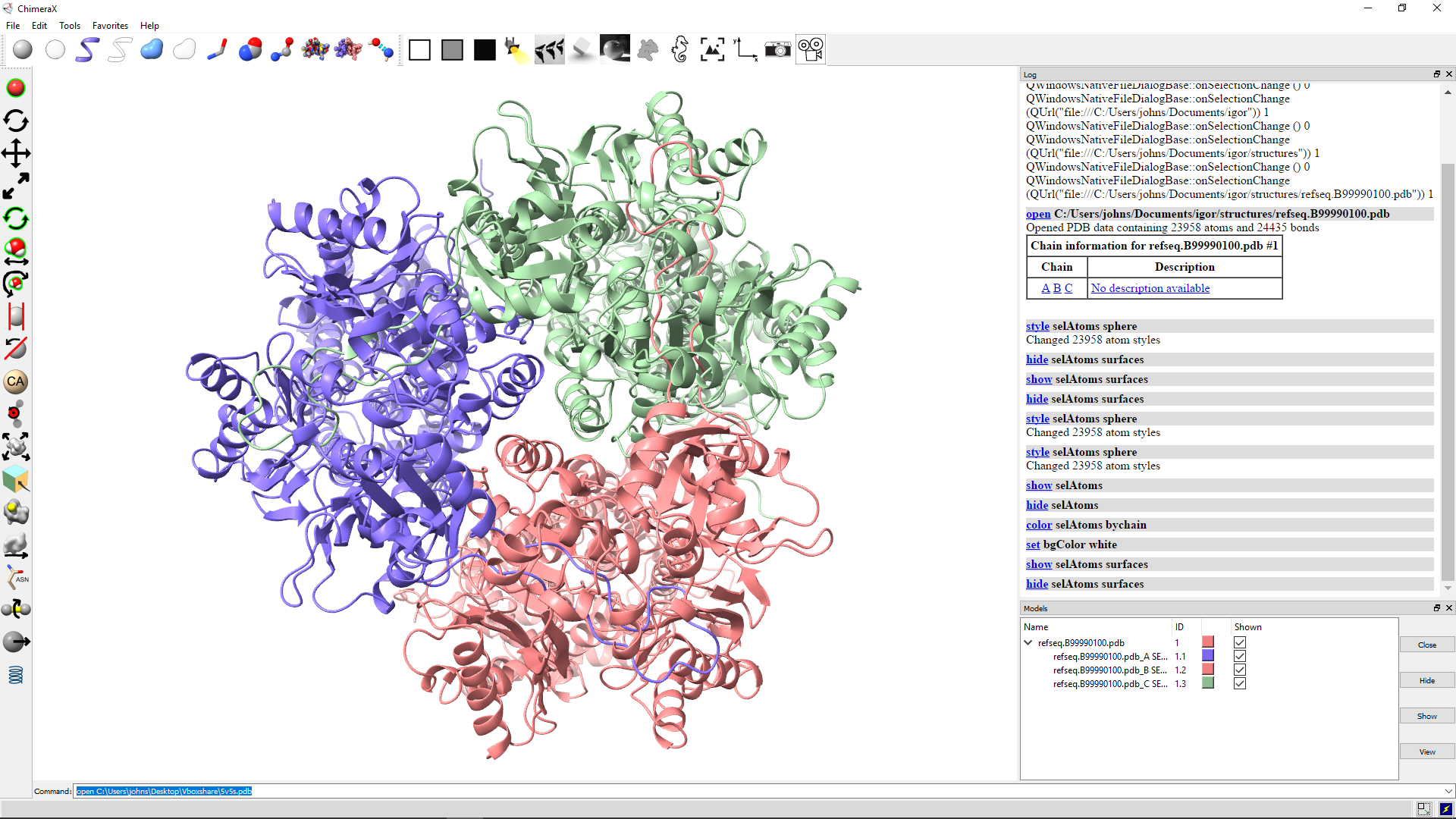


Drug efflux pore

**B**


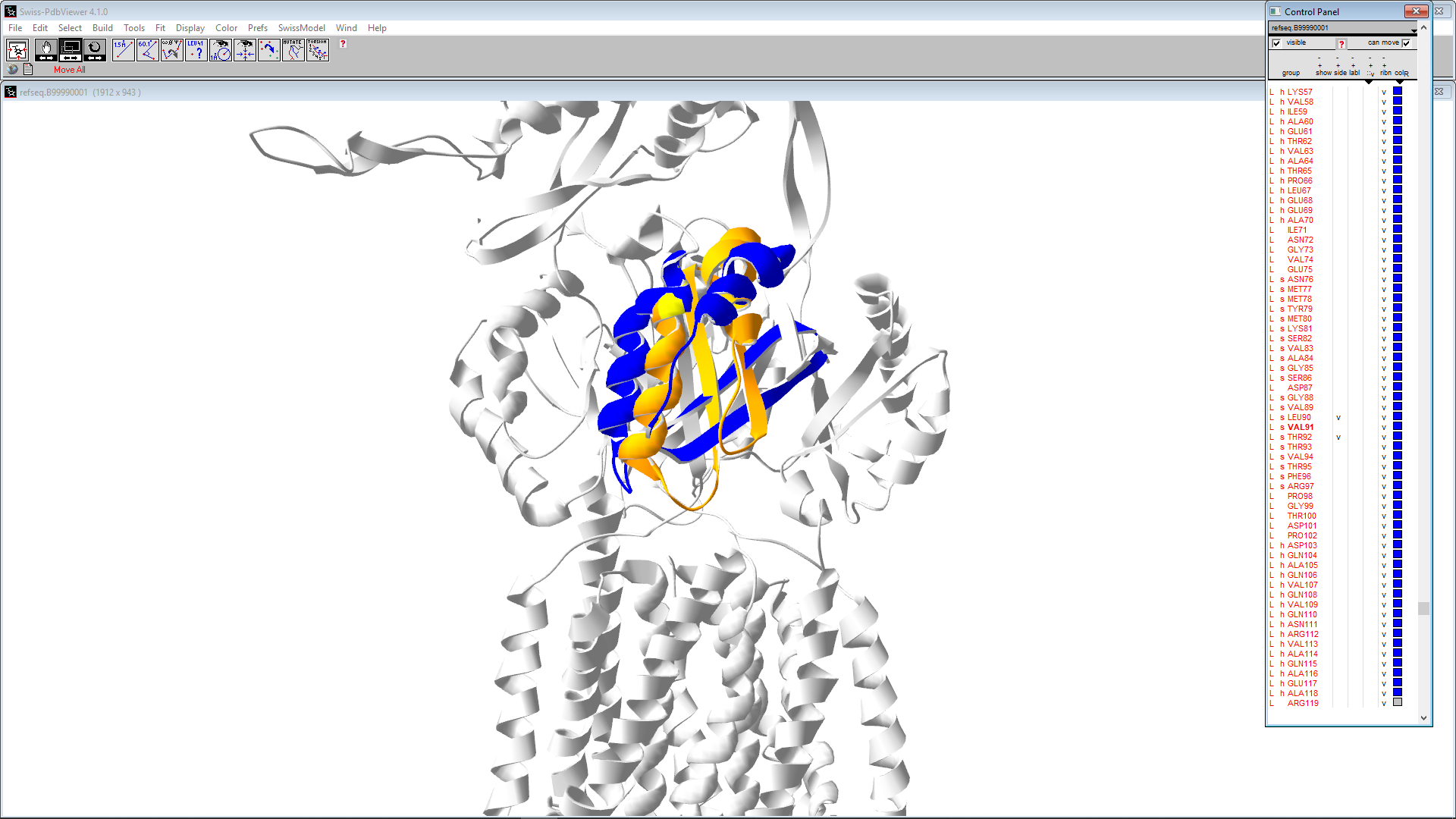


TM domain

**Fig. S6**. The effect of mutations L90I and T92A on the efflux channel.
