# Supplementary material for: Preventive antibiotic treatment of calves: emergence of dysbiosis causing propagation of obese state‐associated and mobile multidrug resistance‐carrying bacteria
Source: Microb Biotechnol. 2019 Oct 30;13(3):669–82. doi: 10.1111/1751-7915.13496 (PMC7111097; doi:10.1111/1751-7915.13496)
Supplement: Supplementary file 2 — Appendix S1 . Experimental procedures. [file MBT2-13-669-s002.docx]

**Appendix S1**

**Experimental procedures**

**Sample preparation**

Freshly passed faeces was collected from medicated and non-medicated animals immediately before the

treatment (T0) and at 3 (T3) and 7 (T7) days after the treatment. In each case, approximately 40g of faeces were collected, then divided equally into four 50 mL Falkon® tubes, re-suspended in 40 mL of cold, sterile phosphate buffered saline (1xPBS, 8 g/L NaCl, 0.2 g/l KCl, 1.44 g/L Na2HPO4 and 0.24 g/L KH2PO4 pH7.4) and centrifuged at 300×g (1,400 rpm) for 10 min at 4°C to remove debris, including undigested food. The bacterial cells were pulled down by centrifugation of the resultant supernatant at 3,000×g (4,400 rpm) for 30 min at 4°C. The pellets of bacterial cells were washed with 45 mL of cold 1xPBS and cleared further by centrifugation at 3,000xg as described above. The faecal debris/pellets from the first spin were washed again with 40 ml of cold 1xPBS and processed as above in order to pull down any co-precipitated cells.

Pellets of bacterial cells were combined, re-suspended in 10 mL of cold 1xPBS and filtered through

Miracloth (Calbiochem®) to clear the cell fraction further. Cells were collected from the flow-through by

centrifugation at 3,000×g for 30 min at 4°C and were stored at -80°C.

**Genomic DNA sequencing**

For six calves, total DNA samples were sequenced at three time points: before treatment and at T3 (day 3) and T7 (day 7) after treatment. The V3 and V4 regions of the bacterial 16S rRNA gene were amplified with standard Illumina primers, forward primer: 5’-CCTACGGGNGGCWGCAG-3’ and reverse primer: 5’-

GACTACHVGGGTATCTAATCC-3’ to produce 550 bp products for sequencing (Vertis, Germany). Sequencing was carried out on an Illumina MiSeq, producing 2x300bp paired-end reads.

For whole (meta) genome shotgun sequencing for antibiotic resistome analysis, gDNA of To and T7 for one non-medicated and two medicated animals were used for sequencing (Vertis, Germany). Sequencing was carried out on an Illumina NextSeq 500, producing 1x75bp single-end reads.

**Semi-quantitative PCR amplification of 16S rRNA and uidA genes**

The uidA gene, encoding the β-glucuronidase (a marker gene for E. coli) was targeted with forward primer, 5’-CGAACTGAACTGGCAGAC-3’ and reverse primer, 5’-AATGCGAGGTACGGTAGG-3’ as described previously (Srinivasan *et al*., 2011) while the 16S RNA gene was amplified with forward primer, 5’-AGAGTTTGATCCTGGCTCAG-3’ and reverse primer, 5’-ATTACCGCGGCTGCTGG-3’. For each assay, 240ng of To and T7 gDNA samples were diluted in a 2-fold series, amplified (3 min at 95°C followed by 15 cycles of 1 min at 95°C, 45 sec at 56°C and 1 min at 72°C) with 10 μM primers, 10 μL of 2xPCR Mastermix (Roche) and analysed on 1% TAE agarose gels. Independent triplicate samples were analysed in parallel.

**PCR, cloning and Sanger sequencing of the mcr-2 and oqxB genes**

240 ng of gDNA was used for the first PCR with 10 μM primers, 10 μL of 2xPCR Mastermix (Roche), 3 min at 95°C and 40 cycles of 1 min at 95°C, 45 sec at 50°C for mcr-2 or 60°C for oqxB and 1 min at 72°C, and a final elongation step for 10 min at 72°C. Nested PCR with 2 μL of the first PCR reactions as the template was performed as described above with the corresponding annealing temperature for each primer set. mcr-2 was targeted with forward primer 5’-CATCACATCACTCTTGGT-3’ and reverse primer 5’-TTGAACTGCACATGGTCA-3’ (5-761 nts of the gene) followed by a nested PCR for the 44-726 nts region with forward primer 5’-GTGCTGATGGGTTTGGTG-3’ and reverse primer 5’- GACGACGAACACCACTAG-3’ at 55oC for annealing. A part of the oqxB gene was amplified with forward primer 5’-CTGGAGGAAGCGATCAAC-3’ and reverse primer 5’-ATTGGGATCCAGCCAGAC-3’ (199-582 nts of the gene) followed by amplification with the nested forward primer 5’-GTCACCACCGTCACCTTC-3’ and the same reverse primer (312-582 nts of the gene) and annealing temperature at 60oC. To confirm the presence of the pOLA52 plasmid in the gDNA of the medicated samples at day 7 nested PCRs were carried out as described for the mcr-2/oqxB gene. The first set of PCRs was conducted with forward primer 5’-TCAAGAGAGCGACCGCTT-3’ and revers primer 5’-TGAACGAGCAGAGCGTGA-3’ at the annealing temperature of 59oC. The nested PCRs targeted the region between 11,464 and 11,700 nts with the same forward primer and reverse primer 5’-CGTCAGGAACTGCTTCAG-3’ with the annealing temperature of 55oC. The PCR products were visualised on 1% agarose gel, extracted from the gel using QIAquick® Gel Extraction Kit (Qiagen), cloned into pGEM®-T Easy Vector using pGEM®-T Easy Vector Systems (Promega) according to the manufacturer’s protocol and transformed into JM109 competent cells (Promega). Ten randomly selected clones for each set were used for Sanger sequencing (GATC, UK).

**Reference**:-

Srinivasan, S., Aslan, A., Xagoraraki, I., Alocilja, E., and Rose, J.B. (2011) Escherichia coli, enterococci, and Bacteroides thetaiotaomicron qPCR signals through wastewater and septage treatment. Water Res

45:2561–2572. https://doi.org/10.1016/j.watres.2011.02.010.
